# Salvaging Digital Replantation and Revascularisation: Efficiency of Heparin Solution Subcutaneous Injection

**DOI:** 10.1155/2018/1601738

**Published:** 2018-11-21

**Authors:** Haz Alfeky, Paul McArthur, Yasser Helmy

**Affiliations:** ^1^Consultant Plastic Surgeon, Plastic Surgery Department, University Hospital Coventry and Warwickshire, Coventry, UK; ^2^Consultant Plastic Surgery, Whiston Hospital, Liverpool, UK; ^3^Professor of Plastic Surgery, Alazhar University Hospitals, Cairo, Egypt

## Abstract

**Background:**

Distal digital replantation and revascularisation remains one of the demanding microsurgical procedures due to the difficulty of vascular anastomosis. Venous congestion is the most commonly encountered problem after replantation due to the difficulty of venous anastomosis in traumatic injuries. Heparin, among other drugs, is commonly used to facilitate venous drainage and prevent thrombosis. However, systemic heparin can be contraindicated in some patients. The senior author has experience of subcutaneous heparin injection for venous congestion in thirteen patients.

**Methods:**

An amount of 1 ml of calcium heparin (25,000 U) was mixed in 2.4 ml of normal saline making a solution that has 1000 U per 0.1 ml. 1000 U (0.1 ml) of the solution was injected directly into the congested replanted digits. This was repeated twice daily until venous congestion improved.

**Results:**

All the congested replanted digits survived without systemic side effects. There were no local side effects of the treatment. The PT and APTT have shown slight increase but they remained within the normal range. Haemoglobin levels have dropped slightly but no patients were at any risk of developing anaemia or needed blood transfusion.

**Conclusions:**

Subcutaneous heparin injections can salvage the replanted digits when venous congestion is a warning flag for replantation failure. It is safe and very efficient in patients where systemic heparin cannot be administered. However, this article shows the results in only thirteen patients which is a small number to show the efficacy, safety, and side effects.

## 1. Introduction

The hand is the most injured organ in our bodies and due to the recent workplace regulations, the incidences of nonfatal workplace amputations have decreased [1].

Recovery of both psychological and physical function requires finger replantation surgery when possible, because successful replantation helps restore the aesthetics of the patient's hand as well as its previous functions [2–5].

Distal replantation is considered to be one of the most challenging surgeries carried out by hand surgeons due to its technical difficulty, time consumption, and outcomes. Despite all of that, over the last few decades there have been successful and promising results with a success rate comparable to the elective free flap procedures.

The very first finger replantation was reported in 1968. Since then, there have been many reports that have shown the steady progress and advancement of the technique [6–8]. Arterial anastomosis is crucial for the survival of the replanted part. This can be augmented by venous anastomosis [5]. However venous insufficiency can happen in replanted digits. If this happens, then necrotic changes could be unpreventable if not treated. Unfortunately, venous insufficiency is more common in replanted tips, in children, and in significant trauma [9, 10].

Studies indicate that using heparin solution in microvascular anastomosis improves the outcomes of the surgery. This is usually achieved through intermittent bolus intravenous injection [4, 6, 11]. Barnett et al. [12] first described using direct heparin injection into the congested replanted fingers managing to save three patients with postreplantation venous congestion. Jeng et al. [13, 14] and Iglesias and Butron [15] have investigated the pharmacokinetics of the subcutaneous heparin injections.

The aim of this study is to review the efficiency of subcutaneous heparin solution injections to improve the venous congestion and hence the outcomes and survival of distal digital replantation in patients who cannot tolerate systemic heparin due to associated comorbidities like gastric ulcers, varices, or haemorrhoids.

## 2. Patients and Methods

Between July 2011 and September 2013, out of 23 finger replantation procedures, 13 patients with Tamai Zone I and Zone II distal amputations were not suitable for systemic heparinization due to history haemorrhoids or gastric ulcer disease (Table 1). Inclusion criteria were as follows: any patient who had distal digital replantation with venous congestion in the first 4 hours after the initial replantation and was not suitable for systemic heparin due to the associated comorbidity and tendency to bleed. Exclusion criteria were as follows: congestion more than 4 hours and patients suitable for systemic heparin solution administration. Initial replantation was done with standard arterial microvascular anastomosis followed by venous anastomosis. Dorsal veins were primarily used to establish the venous drainage. However, the volar veins were used to establish the venous drainage in Tamai Zone I due to the difficulty to find suitable dorsal veins. Postoperatively, the digits were monitored for any ischaemia or congestion. If any venous insufficiency was noticed, then revision surgery was performed within 4 hours. If this failed, then heparin infusion was subcutaneously injected into the replanted part through a small skin incision.

An amount of 1 ml of calcium heparin (25,000 U) was mixed in 2.4 ml of normal saline making a solution that has 1000 U per 0.1 ml. 1000 U (0.1 ml) of the solution was injected directly into the congested replanted digits. This helped in bleeding from the replanted part and helped to improve the congestion for 6 hours. That was followed by 2 doses in the next 24 hours and then twice daily for 3 to 5 days (mean 4 days: 7.5 injections mean) until it was clear clinically that the replanted part has developed collateral circulation and established its own venous drainage with subsequent congestion improvement or it became clear clinically that the replanted part did not survive with visible infarcts and necrosis.

## 3. Results

Out of the 13 patients who received subcutaneous calcium heparin for postreplantation venous insufficiency, six patients have started to receive the heparin on the same revision surgery day, five on day 1 postoperatively, and two on the 2nd postoperative day. The patients have received 6-10 doses of heparin with the mean of 7.3. Bleeding continued for 3 to 6 hours, with a mean duration of 4.3 hours after the first dose of 1000 units. The bleeding continued for a shorter period after the 2nd dose ranging from 2.5 to 5 hours with the mean duration of 3.6. The third and all the subsequent doses have nearly the same effect with mean duration of 3.4 hours (Table 2).

Prothrombin time has shown a slight prolongation with a preheparin range of 9.5 to 13 second with the mean of 10.6 seconds. This has increased to a range of 9.6 to 13 second with a mean of 11.1 second. The APTT mean was 35.9 with a range of 30 to 40 seconds before heparin treatment. This has increased slightly to a mean of 37 seconds with a range of 32 to 40 seconds.

To monitor the blood loss because of the heparin injections, haemoglobin levels were measured in all patients. The haemoglobin level before the treatment ranged from 11 to 16.2 with a mean level of 13.2. This has dropped slightly to a range of 10.5 to 16.1 with the mean of 13 showing very slight decrease due to the minimal bleeding despite the high concentrated heparin solution (Table 3).

Patients did not develop any bleeding from the haemorrhoids or the gastric ulcer. They did not need to change any of their medications for these conditions. No blood transfusions were required due to the treatment with the heparin and the patients did not have to stay any longer than expected. All the replanted parts survived apart from one case which had partial necrosis of the tip of the finger. This was managed conservatively without any need for theatre revisits.

**Figure 1:**
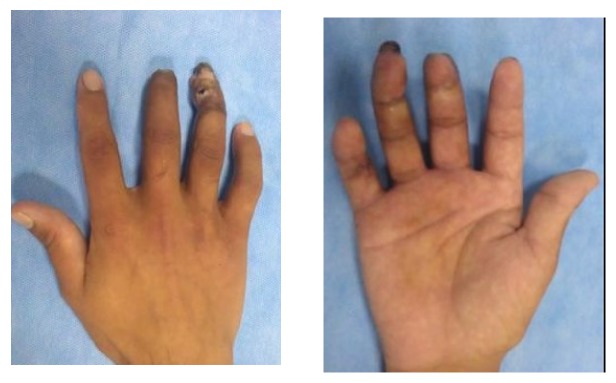


**Figure 2:**
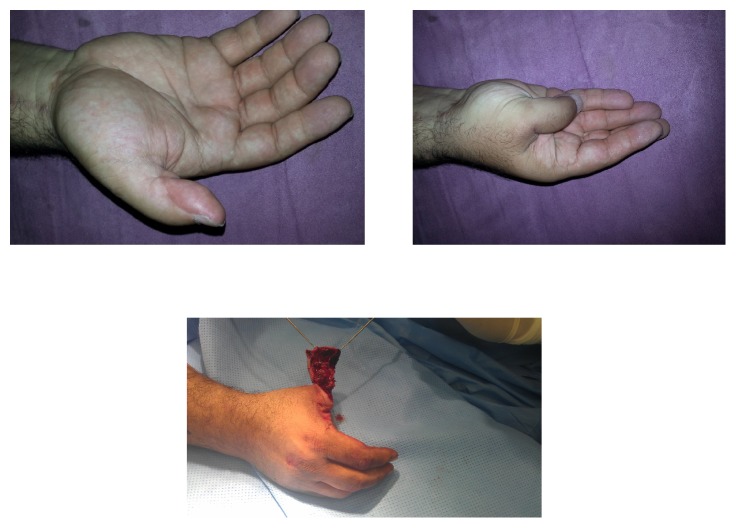


All patients had 4 to 8 months follow-up (mean 6.4 months). Six patients were monitored for more than 6 months (Figures 1 and 2) and they had altered sensation and early signs of cold intolerance. Three of them have received desensitisation treatment to improve their symptoms.

## 4. Discussion

Tamai has divided distal digital amputations into Zone I and Zone II according to the level of amputation with Zone I extending from the fingertip down to the base of the nail and Zone II from the base of the nail to the DIP joint [16].

Microsurgeons can treat venous congestion by either systemic heparin [8, 17], aspirin [18, 19], a combination of both, dipyridamole [6, 17], low molecular weight heparin [19], low molecular weight dextran [20, 21], urokinease [20, 22], or heparin-soaked gauze topically or leeches [23–26].

Despite the efficiency of systemic heparinization and aspirin to manage congestion and thrombosis, they could be contraindicated in some situations where there is a risk of bleeding like in haemorrhoids, liver disease, and active gastric ulcers [15, 27].

Heparin soaked gauze is easy to apply, cheap, and easy to teach to nursing staff which facilitate the early postoperative care. However, it easily dries, sticks to the wound, and becomes painful. Observation of the digit is more difficult as well. This is because of the moist softened keratin by the heparinised saline [28].

Most of the heparin preparations nowadays are calcium heparin due to its higher tissue affinity than sodium heparin preparations. Subcutaneous calcium heparin has shown to have longer biological activity than intravenous heparin [28]. Iglesias et al. showed that the effect of subcutaneous calcium heparin, when injected into an amputated digit, can persist for nearly 24 to 48 hours [15].

Hirudo Medicinalis, the medical leeches working through sucking the congested replanted finger as well as inducing bleeding through secretion of hirudin which was first extracted in 1957 by Markwardt and a protease inhibitor found in Hirudo medicinalis salivary glands, is a potent inhibitor of thrombin [29]. Despite Bivalirudin (synthetic analogue of hirudin) being 10 times more potent than argatroban, which is a selective antithrombin agent, it cannot be used subcutaneously [30].

Leeches are easy to apply, reliable, and cheap [26, 31]. They have shown success rates between seventy percent and one hundred percent [14, 24]. However, persistent bleeding, bacterial infection, anaphylactic shock, and allergic reactions are reported side effects as well as patient refusal occasionally. Antibiotic coverage with gentamycin is often required while treating with the leeches. Blood transfusion also has been reported as a consequence of uncontrolled action of the leeches [32, 33].

Jeng et al. injected the congested facial amputation with heparin saline solutions [13]. They reported the use of this technique in 1994 in 2 facial amputation cases [14].

Iglesias et al. used the same technique in 1999 in Mexico for congested amputated digits in 3 patients [15]. In 2007, Yokoyama et al. uniquely used the heparin saline subcutaneous injection to treat seven cases of post-finger replantation venous congestion where systemic sodium heparin was contraindicated, thereby avoiding bleeding complications [28].

The limitations of this study though include the small group of patients who fitted into the inclusion criteria and the lack of definitive conclusion that the reason of increased survival was purely due to the heparin injection. For these reasons, we would recommend that the indications, protocol, and outcomes of this approach are not confirmed yet and it will be necessary in the future to establish an agreed protocol based on the outcomes of prospective studies which may or may not support its efficiency.

## 5. Conclusion

The subcutaneous injection of heparin is easy, cheap, cost effective, and reliable treatment for postreplantation venous congestion where systemic heparin will be contraindicated.

## Figures and Tables

**Table 1:** Patient's data: age, sex, mode if injury, digit affected, level, and comorbidities.

	**Age**	**Sex**	**Mode**	**Digit**	**Tamai Zone**	**Associated morbidity**
**Patient 1**	24	M	Knife Laceration	Left Index, Incomplete	I	Haemorrhoids

**Patient 2**	35	M	Knife laceration	Left little, complete	II	Haemorrhoids

**Patient 3**	26	F	Circular saw	Right Ring, Incomplete	II	Haemorrhoids

**Patient 4**	19	M	Blender	Left Index, complete	II	Haemorrhoids

**Patient 5**	62	F	Knife laceration	Left Middle, Incomplete	I	Haemorrhoids, Gastric ulcer

**Patient 6**	51	F	Circular saw	Right Index, complete	II	Gastric ulcer

**Patient 7**	35	M	Circular saw	Left little, Complete	I	Gastric ulcer

**Patient 8**	42	F	Knife laceration	Right Index, Incomplete	II	Gastric ulcer

**Patient 9**	28	F	Blender	Left Index, Incomplete	II	Haemorrhoids

**Patient 10**	52	M	Circular saw	Right thumb, Incomplete	I	Gastric ulcer

**Patient 11**	50	M	Blender	Right Ring, Incomplete	II	Haemorrhoids, Gastric ulcer

**Patient 12**	36	M	Circular saw	Left Index, Incomplete	II	Gastric ulcer

**Patient 13**	49	M	Knife laceration	Left Thumb, Incomplete	II	Gastric ulcer

**Table 2:** Timing of the first heparin dose, Duration of bleeding obtained after the 1^st^ and 2^nd^ injections in hours, total number of injections, and the results.

	1^**st**^** dose timing**	**Bleeding after **1^**st**^** dose (Hr)**	**Bleeding after **2^**nd**^** dose (Hr)**	**Total number of injections**	**Result**
**Patient 1**	Same day	6	4	6	Success

**Patient 2**	Day 1	4	4	8	Success

**Patient 3**	Same day	3.5	3.5	6	Success

**Patient 4**	Same day	4.5	4	7	Success

**Patient 5**	Day 1	5	3	6	Success

**Patient 6**	Day 2	4.5	2.5	10	Success

**Patient 7**	Same day	6	5	10	Success

**Patient 8**	Day 1	5.5	5	7	Success

**Patient 9**	Same day	4	4	8	Success

**Patient 10**	Day 1	3	2.5	6	Success

**Patient 11**	Day 2	3.5	3.5	9	Part. necrosis, managed conservatively

**Patient 12**	Day 1	3.5	3	9	Success

**Patient 13**	Same day	4	3.5	6	Success

**Mean**		4.3	3.6	7.3	

**Table 3:** Effect of heparin subcutaneous injection on the bleeding parameters: activated partial thromboplastin time (APTT), prothrombin time (PT), and haemoglobin concentration (Hb%).

	PT		APTT		HB%	
	Before	After	Before	After	Before	After
Patient 1	9.7	9.7	30	32	12	12

Patient 2	10.2	10.5	35	35	11	10.5

Patient 3	11	11	37	38	14	13.8

Patient 4	13	12.6	40	40	13.5	13.5

Patient 5	11	11	37	36	11.5	11.4

Patient 6	10	10.5	36	37	16.2	16.1

Patient 7	9.5	9.6	34	34	11.4	11

Patient 8	9.8	11	35	40	12	12

Patient 9	12	12.8	38	37	15.2	15

Patient 10	12	12	38	39	14	14

Patient 11	10	13	36	40	16	15.4

Patient 12	9.5	10	34	35	13.5	13

Patient 13	10.5	10.5	37	38	11.9	11.7

Mean	10.6	11.1	35.9	37	13.2	13

## Data Availability

The data used to support the findings of this study are included within the article. If any further details are required, the authors are happy to provide them upon written request to the ethics committee of Alazhar University, Cairo.
